# Properties of Chemically Modified (Selected Silanes) Lignocellulosic Filler and Its Application in Natural Rubber Biocomposites

**DOI:** 10.3390/ma13184163

**Published:** 2020-09-18

**Authors:** Justyna Miedzianowska, Marcin Masłowski, Przemysław Rybiński, Krzysztof Strzelec

**Affiliations:** 1 Institute of Polymer & Dye Technology, Lodz University of Technology, Stefanowskiego 12/16, 90-924 Lodz, Poland; justyna.miedzianowska@dokt.p.lodz.pl (J.M.); krzysztof.strzelec@p.lodz.pl (K.S.); 2 Institute of Chemistry, Jan Kochanowski University, Żeromskiego 5, 25-369 Kielce, Poland; przemyslaw.rybinski@ujk.edu.pl

**Keywords:** modification, silanization, cereal straw, natural fibres, elastomer composites, biocomposites, natural rubber, functional properties, flammability

## Abstract

This article concerns the functional properties of elastomeric composites reinforced with modified lignocellulosic material obtained from cereal straw. The aim of the research was to acquire new knowledge on the effectiveness of cereal straw modification methods in multifunctional properties, while reducing the flammability of newly designed elastomeric materials made of natural rubber. The article deals with investigating and explaining dependencies that affect the performance and processing properties of polymer biocomposites containing modified cereal straw. Three different silanes were used to modify the lignocellulosic filler: n-Propyltriethoxysilane, Vinyltriethoxysilane, and 3,3′-Tetrathiobis(propyl-triethoxysilane). The influence of the conducted modifications on the morphology and structure of straw particles was investigated using a scanning electron microscope, contact angle measurements, and thermogravimetric analysis technique. The increase in hydrophobicity and thermal stability of natural fibers was confirmed. In turn, the impact of silanization on the properties of filled composites was determined on the basis of rheometric characteristics and cross-linking density, static mechanical properties, tear resistance, thermal stability, and flammability tests. Noteworthy was the improvement of the mechanical strength of biocomposites and their resistance to burning. Correlations affecting the structure, morphology, dispersion, and properties of produced composites can facilitate the indication of a further research path in the field of development of new elastomeric biomaterials.

## 1. Introduction

Significant intensification of research work on composites observed in recent years has focused on natural fiber reinforced polymer composites (NFPCs). When designing composite materials, in particular with biofillers, the selection of a suitable polymer matrix is an extremely important issue. Thermoplastics widely used in fiber technology are polyethylene (PE) [1], polypropylene (PP) [2], and polyvinyl chloride (PVC), while phenolic, polyester, and epoxy resins are mostly utilized thermosetting matrices [3]. Current trends in the development of biocomposites are also focused on materials made of biodegradable polyesters: PHB—poly (hydroxybutyrate), PHV—poly (hydroxyvalerate), PLA—poly (lactic acid), or PCL—poly (ε-caprolactone) [4]. More often, not only new biopolymers, but also renewable fillers are being sought as alternative sources that will allow them to receive environment-friendly functional biocomposites. The plants that produce cellulose fibers can be classified into bast fibers (jute, flax, ramie, hemp, and kenaf), seed fibers (cotton, coir, and kapok), leaf fibers (sisal, pineapple, and abaca), grass and reed fibers (rice and corn), and core fibers (hemp, kenaf, and jute), as well as all other kinds (wood and roots) [5,6].

However, the effect of fast biodegradation of polymers is often not desirable because of the time of use and the lack of recycling possibilities. A poorly understood and used polymeric matrix for the production of biocomposites is elastomers, for example, natural rubber. The aim of the work is to use natural rubber (NR) as a possible natural polymer matrix in those types of composites. NR is obtained from the rubber tree (Hevea brasiliensis) in the form of latex. NR (cis-1,4-polyisoprene) is important as it possesses the general features of other rubbers in addition to characteristics like high gum tensile strength and good tackiness. NR is produced by plants, which means it is renewable, inexpensive, and creates fewer health hazards [7].

From a research and cognitive point of view, cereal straw powder can be considered as a new and as yet not fully characterized bio-additive to elastomer composites made of natural rubber. Straw is produced throughout the world in enormous quantities as a by-product of cereal cultivation. Cereal straw (CS) from wheat, oats, barley, rye, triticale, and corn, left after harvesting, forms a large source of fibrous biomass. Plant cell walls of straw are constructed from a combination of a variety of compounds that can be generally grouped into cellulose, lignin, hemicelluloses, and pectin and whose relative proportions depend on the plant species, specific tissue, and growth stage [8]. The main fractions of CS are nodes, internodes, and leaves [9]. The biochemical composition of straw is characterized by a typical composition of an agricultural-based lignocellulosic residue: it contains on average 30–45% cellulose, 20–25% hemicellulose, and 15–20% lignin, as well as a number of minor organic compounds. Straw is poor in nitrogen, but relatively high in inorganic compounds [10].

Individual straw fibers have a much lower tensile strength and lower compressive strength as compared with other fibers that are added to polymers. A limited number of studies have reported the use of straw fibers for composite production [11]. For this reason, it is important to know and examine another form of straw, in this case, the powder.

The interest in biocomposites is growing rapidly both in terms of their use in industry and in basic research. It is mainly caused by legal regulations in many European countries towards the reuse and recycling of polymeric materials, as well as a reduction of production costs and the use of cheap, natural, and renewable plant materials. Composites reinforced with natural fibers are environmentally friendly and are applied in many branches of industry, such as automotive, transport, construction, packaging, and household articles production. Wide possibilities of using these composites, especially in construction and transport, forced to introduce appropriate functional properties like mechanical, damping, barrier, and thermal properties. In order to achieve a sufficiently high application level of biocomposites, it is required to obtain very good adhesion at the polymer–natural fiber interface. In order to improve the polymer–filler interactions, many chemical modifications are used, including treating the surface of the filler with a modifier or adding a coupling agent to the composite. Silanes are widely investigated compounds for improving interfacial adhesion. These tests are usually carried out for typical fillers of polymer composites, that is, silica [12], carbon black, and carbon nanofibers [13]. The effect of the modification of natural fibers with silicon compounds on the properties of polymer biocomposites is less known, especially in the case of rubber. Several research studies attempted to improve compatibility between natural fiber and rubber using the addition of compatibilizer or fiber surface modification [14,15,16].

This article deals with an interesting aspect of polymer composites technology, the effect of adhesion between straw and natural rubber. In order to increase the interactions at the elastomer–filler boundary, straw modifications were applied. By modifying the surface of filler particles with compounds similar in structure to rubber macromolecules or able to react with them, better interfacial compatibility could be achieved.

The main aim and novelty presented in the article was to show and compare the influence of modified straw fibers with specially selected silanes on the properties of elastomeric biocomposites. Silanes containing different species can show variable activity in the elastomer cross-linking process. The modifications of compatibilizing agents used so far in studies on polymer biocomposites mainly included modification of the lignocellulosic material for which the matrices were thermoplastics, such as polypropylene (PP) and polyethylene (PE) [17]. Natural rubber has completely different material features, characteristics of the spatial structure, and manner of interactions with the filler. In research on polymeric composites, the use of an appropriate medium is a key issue and forms the basis of its subsequent, relevant properties. The selection of silanes is crucial, particularly the choice of their chemical structure. Moreover, in combination with straw and natural rubber, they are an element of innovation in the study of this type of materials. Furthermore, the different activities of modifiers in the process of polymer cross-linking could serve to determine the impact of interactions between the elastomer and straw in the context of the functional properties of composites. Modification of fibers could lead to the improved degree of dispersion and distribution of the filler in the matrix as compared with non-surface treated powders. The structure of the composite also plays a significant role in its thermal stability [18].

Homogeneously distributed filler particles create a barrier by limiting the diffusion of gases and gaseous thermal decomposition products, and thereby increase the thermal stability of the composites. For example, the problem of increasing the thermal stability and reducing the flammability of rubber products is of particular importance because of serious health and life hazards, as well as pollution of the natural environment caused by the emission of large amounts of evolved smoke and toxic substances during their thermal decomposition. The polymers used to make composites, including NR, are usually flammable. At high temperatures, the polymers undergo thermal decomposition. Initially, high temperatures, smoke and volatile substances are generated. The latter consists of a mixture of flammable volatile substances such as monomers, hydrocarbons, carbon monoxide, and non-flammable gases, including carbon dioxide. Undoubtedly, a key issue in terms of the use of lignocellulosic raw materials as bio-fillers for polymers is the radical limitation of their flammability. The literature review shows that the flammability of natural fibers largely depends on their chemical composition. Even a small amount of lignin (10%) increases the resistance of natural fibers to fire. The research carried out by Kozłowski and colleagues clearly shows that natural fibers rich in lignin (flax, hemp) are characterized by a lower rate of heat released than fibers with a high cellulose content. This shows the ability of lignin to catalyze carbonization reactions, which positively affect the formation of an insulating carbon layer, increasing the fire resistance of polymer bio-composites containing them [19,20]. The increase in the carbonization reaction efficiency during the decomposition of natural fibers can be obtained by subjecting them to chemical modification. In the subject literature, it is possible to find a few articles on fire retarding natural fibers. For example, Kondola and Horrocks modified cellulose (viscose) as well as cellulose fabric using both melamine and phosphor flame retardants. On the basis of the conducted research, they found that a significant amount of carbon generated above 500 °C improves the thermal properties of the modified fibers, as evidenced by both the increase in their activation energy Ea and the reduction of thermal conductivity [21]. Flambard et al. studied flame-retardant flax and wool, intended for the production of fabrics used in public transport, mixing them with PPTA (poly (p-phenylenediamine terephthalamide). The modified fibers were characterized not only by high resistance to fire, but also resistance to the action of UV light, satisfactory mechanical parameters, biodegradability, and easy processing [22]. Suardana et al. modified coir and jute fibers using diammonium phosphate (DAP) and then incorporated them into a thermoplastic polymer matrix. On the basis of the tests performed, they found that the improvement of the bio-composites resistance to fire was directly proportional to the amount of DAP fibers used for modification, which resulted directly from the amount of carbonaceous carbon produced above 500 °C [23]. There is no information in the literature on the subject of chemical modification of lignocellulosic fibers with silicon compounds.

The previous research and experience of the authors [24,25,26] show that the optimal degree of filling for biocomposites with the addition of straw material is about 10–20 phr (depending of the filler type), in order to maintain functional properties at a sufficiently high level. Above 30 parts by weight, performance parameters, especially the material strength, deteriorate.

## 2. Materials and Methods 

Pure cereal straw (a mixture of different types of cereals) was harvested from local polish farms. After drying in a laboratory dryer (70 °C, 24 h), straw was ground into a fine powder using a ball mill Pulverisette 5 Classic Line planetary ball mill (Fritsch, Idar-Oberstein, Germany) for 3 h.

Silanization was carried out by reacting the lignocellulosic material with selected silanes: n-Propyltriethoxysilane, Vinyltriethoxysilane, and 3,3′-Tetrathiobis(propyl-triethoxysilane (Sigma-Aldrich, St. Louis, MO, USA). Each modification was carried out in a solution of water and ethanol (water to ethanol ratio = 1:4) by adding straw and silane in the ratio of 10:1. The 1 L reaction volume was first treated in ultrasounds for 2 h. Then, the solvent was evaporated using Heidolph, Laborata 4001 (Teltow, Germany) efficient rotary evaporator and the residue (silanized straw) was dried at 70 °C to achieve constant mass.

Natural rubber (NR)–RSSI, cis-1,4-polyisoprene, with a density of 0.93–0.98 g/cm^3^ was provided by Torimex Chemicals (Lodz, Poland). This elastomer was vulcanized with sulfur (Siarkopol, Tarnobrzeg, Poland), microsized zinc oxide (ZnO, Sigma-Aldrich, St. Louis, MO, USA), and stearic acid (SA, Avantor Performance Materials, Gliwice, Poland) as the standard activators and 2-mercaptobenzothiazole (MBT, Sigma-Aldrich, St. Louis, MO, USA) as an accelerator.

Composition of typical elastomer mixture: NR rubber (100 phr (parts per hundred rubber)); sulfur (2 phr); mercaptobenzothiazole (2 phr); zinc oxide (5 phr); stearin (1 phr); and fillers—0 (reference sample), 10, 20, and 30 phr (biocomposites).

Thermogravimetric analysis (TG) was applied to study the thermal stability of natural fillers and the effect of lignocellulose fillers on the thermal stability of natural rubber vulcanizates. A Thermogravimetric Analyzer TGA/DSC1 (Mettler Toledo, Greifensee, Switzerland), previously calibrated with indium and zinc as standards, was used for measurements. The analysis of samples was performed using a two-step procedure. Test specimens of approximately 10 mg were placed in aluminum oxide crucible and heated from 25 °C to 600 °C in an argon atmosphere with a heating rate of 10 °C/min. Next, the gas was changed into the air (flow rate = 50 mL/min) and heating was continued up to 700 °C with the same heating rate. In the case of vulcanization, the second step of heating was continued up to 900 °C.

The lignocellulosic material was pressed into pellets to measure the contact angle. The static advancing contact angle between a water droplet (10 µL) and the pressed fiber disc was measured using the sessile drop method on a goniometer (DataPhysics, OCA 15EC, San Jose, CA, USA). The contact angle was measured from the captured images.

The morphology of the treated and untreated lignocellulosic material and its biocomposites was tested by scanning electron microscopy (SEM), LEO 1530 Gemini (Zeiss, Oberkochen, Germany) equipped with an energy dispersive spectrometer (EDS) (Zeiss, Oberkochen, Germany). The EDS method was used to perform an elemental analysis of micro-areas of some fillers. Before the SEM–EDS measurement, the samples of filler and fractures of vulcanizates were coated with a carbon target using the Cressington 208 HR system (Watford, England).

Rubber mixtures filled with silanized and untreated straw were prepared in two stages. First, the natural rubber and filler were mixed using an internal mixer N50 Brabender (Duisburg, Germany). Parameters of the process: temperature—50 °C; rotor speed—40 rpm; time of the process—10 min. Then, the curing agents were added to each of the mixtures using a rolling mill. 

The rheometric properties and curing time of rubber mixtures were examined using a rotorless rheometer model (MDR) (Alpha Technologies, Bellingham, WA, USA) at 160 °C, according to standard ISO 6502.

The vulcanization kinetics of elastomer blends was investigated using the differential scanning calorimetry (DSC) technique. Measurements were performed using a DSC1 calorimeter (Mettler Toledo, Greifensee, Switzerland). The results of the study allowed the determination of the temperature range in which the cross-linking process took place and the enthalpy of natural rubber curing reactions. Parameters of measurement: temperature from −100 to 250 °C, heating rate—10 °C/min, and nitrogen—80 mL/min was used as the protective gas, whereas liquid nitrogen was applied to cool the sample before the measurement.

The polymer mixtures were cured at 160 °C and 15 MPa pressure for a time corresponding with the optimal vulcanization time obtained from rheometer (t_90_). The specimens were formed using steel molds heated by an electrically hydraulic press.

The cross-linking densities (υ_e_) of filler-reinforced rubber composites were determined by an equilibrium swelling test, performed in toluene solvent at room temperature, based on the Flory–Rehner equation (Equations (1) and (2)) [27].

(1) $$\upsilon_{e}=\frac{\ln\left(1-V_{r}\right)+V_{r}+{µV}_{r}^{2}}{V_{0}\left(V_{r}^{\frac{1}{3}}-\frac{V_{r}}{2}\right)}$$

where $\upsilon_{e}$—the cross-linking density (mol/cm^3^); V_0_—the molecular volume of solvent (106.7 cm^3^/mol); and µ—the Huggins parameter of the rubber-toluene systems, given by the following Equation (2):

Μ = 0.478 + 0.404∙V_r_ (2)

where V_r_ is the volume fraction of elastomer in the swollen gel (Equation (3)):

(3) $$V_{r}=\frac{1}{1+Q_{w}\frac{\rho_{r}}{\rho_{s}}}$$

where Q_w_—equilibrium swelling reduced by the filler content (x [phr]) − Q_w_ = (100 + x/100); ρ_r_—density of rubber (g/cm^3^); and ρ_s_—density of solvent (g/cm^3^).

A Zwick Roell 1435 (Ulm, Germany) universal testing machine equipped with an extensometer was applied to measure the tensile properties of vulcanizates. The measurement was carried out for five dumbbell-shaped samples at a crosshead speed of 500 mm/min following standard ISO 37.

The tear test was performed as per ISO 34 for three samples of each composite using the Zwick testing machine (Ulm, Germany) with the test speed of 50 mm/min. The specimen was cut to 100 mm × 15 mm × 1 mm, in a “trousers” shape, with a pre-cut of 40 mm at the center.

The flammability of straw was examined using the FAA micro-calorimeter from Fire Testing Technology Limited (East Grinstead, UK). The temperature of the pyrolyzer was 600 °C, while that of the combustor was 900 °C.

The natural rubber composites were examined using a cone calorimeter from Fire Testing Technology Ltd. (East Grinstead, UK). Elastomer samples with dimensions of (100 × 100 ± 1) mm and thickness of (2 ± 0.5) mm were tested in a horizontal position with a heat radiant flux density of 35 kW/m^2^.

## 3. Results and Discussion

The silane coupling agent has been widely used to enhance the interactions between fillers and polymer matrix. The bi-functional molecular structure of silane was the key to enhance the interactions. Thus, silanes with various type of functional groups have been studied to improve the interfacial adhesion between fillers and polymer [28].

As straw surface modifiers, silanes of the different structures have been investigated, which exhibited different activities in the vulcanization of NR. Vulcanization is a chemical process for cross-linking unsaturated polymeric chains of rubber using sulfur. This process has played an important role to achieve the elasticity of rubber.

The silane structures used in this work are shown in Table 1.

**Figure 1:**
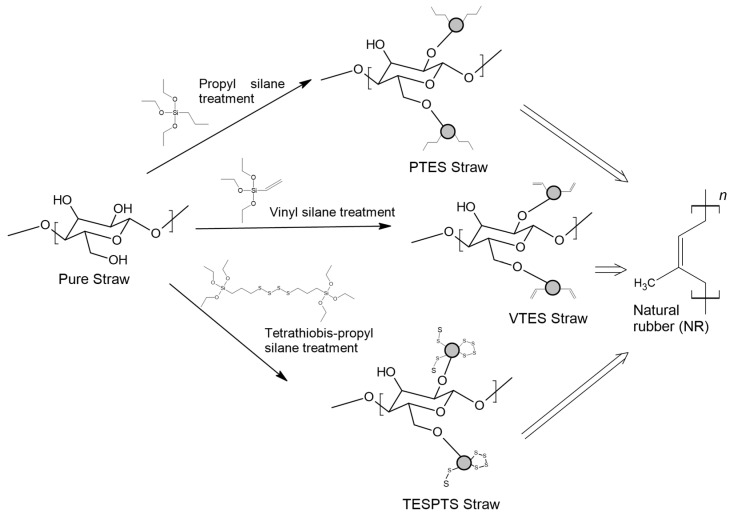
The reaction schemes of silane treatments.

Propyltriethoxysilane (PTES) has been considered as a reference for a non-reactive treatment during the vulcanization of the rubber. However, the surface was becoming hydrophobic and better compatibility was observed with hydrophobic NR. Vinyltriethoxysilane (VTES) was used as a reactive site that contains a vinyl group. A double bond was proposed to react during cross-linking with NR. 3,3′-Tetrathiobis(propyl-triethoxysilane) (TESPTS) was a silane containing sulfur that provides free sulfur for cross-linking with rubber during the vulcanization at a high temperature. The reaction schemes of silane treatment were proposed in Figure 1.

### 3.1. Thermal Analysis of Fillers

**Figure 2:**
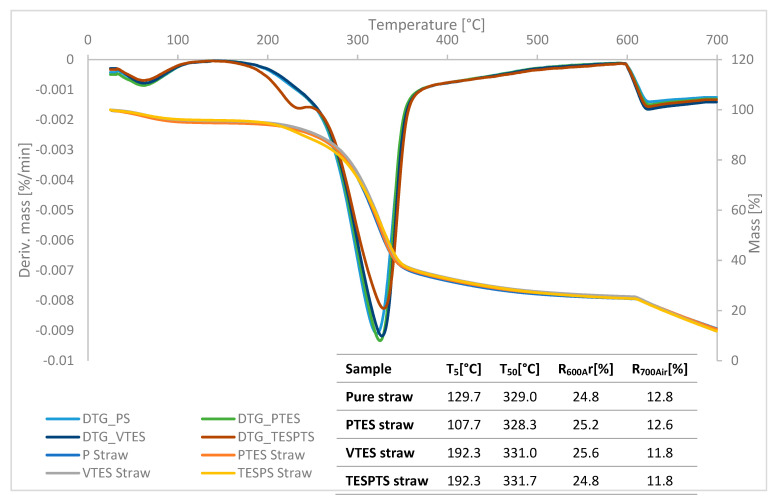
The TGA curves and the first derivative of the TGA curves (DTG curves) of modified and unmodified straw fibers.

Figure 2 shows the thermogravimetric curves (TGA curves) of lignocellulosic fillers (pure straw, straw modified with propyltriethoxysilane (PTES straw), straw modified with vinyltriethoxysilane (VTES straw), straw modified with 3,3′-Tetrathiobis(propyl-triethoxysilane) (TESPTS straw)) and a summary of the characteristic parameters recorded during the TGA analysis (T_5_—temperature of 5% weight loss, T_50_—temperature of 50% weight loss, R_600Ar_—residues after the pyrolysis, R_700Air_—residues in 700 °C).

The analysis of first derivative of the TGA curves (DTG curves) allows distinguishing several characteristic stages of the process. The first stage in the range of 50–120 °C corresponded to the evaporation of water contained in the straw fibers. The greatest weight loss due to moisture removal was recorded for pure straw and PTES straw. This means that these materials were characterized by the highest hygroscopicity. The material treated with VTES and TESPTS showed a reduction in moisture content, possibly due to a reduction in the number of hydroxyl groups after silanization [29]. Besides, the lower water content in the silane-treated straws could be related to the penetration of silane into the cell wall of the lignocellulosic material through the pores and its deposition in the interfibrillar area, consequently creating a barrier preventing water penetration into the straw [30].

The second step is the proper degradation of the lignocellulosic material, which was observed from 170 to 550 °C. Its course depended on the type of straw modification used. Again, the process was fastest for the PTES processed material and the untreated straw. On the other hand, a significant slowdown in thermal degradation was observed for the modified TESPTS filler. From 600 °C, the test atmosphere was changed and the materials were burned in an air atmosphere.

The temperature values at which a 5% weight loss was noted for the VTES straw and TESPTS straw fillers were exactly 192.3 °C and were significantly higher than the temperature recorded for pure straw (129.7 °C) and PTES straw (107.7 °C). This means that the action of vinyltriethoxysilane and 3,3′-Tetrathiobis (propyl-triethoxysilane) improved the thermal stability of these additives. In the case of the analysis of the T_50_ values, the differences were not so significant. Nevertheless, again for the fillers VTES straw and TESPTS straw, the values obtained were the highest. Residues after the pyrolysis process oscillated on a similar level for all types of straw and ranged from 24.8% to 25.6%. Moreover, the values of the residue after incineration did not differ significantly, ranging from 11.8 to 12.8%.

### 3.2. Morphology of the Fillers 

**Figure 3:**
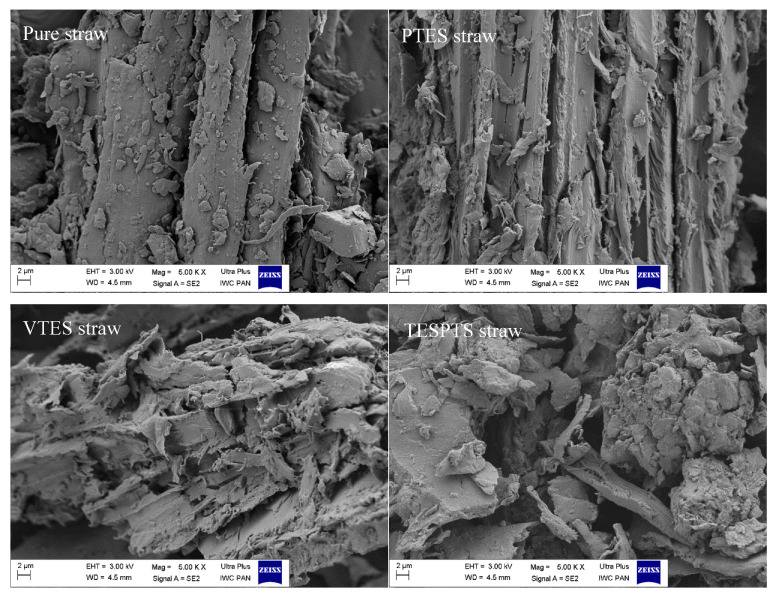
SEM images of ground straw (modified and unmodified) under magnification 5000×.

In order to investigate the effect of the performed silanization on the structure of the fillers, SEM images of untreated and treated straw with PTES, VTES, and TESPTS (Figure 3) were prepared.

Comparing the presented images, it was observed that the straw particles that were formed after grinding were irregular and had different sizes and shapes. Nevertheless, most of the particles were similar in shape to fibers. The surfaces of the pure straw particles were smooth and the cell walls were compact. Modification with silanes significantly influenced the morphology of the fiber. Regardless of the modifier used, the fiber surfaces have become rougher and more jagged. The modifications resulted in cracking of the cell walls and the formation of microcracks on the surface of the fibers. When VTES and TESPTS were used, the lignocellulosic material no longer took the shape of fibers, but rather irregularly shaped finer particles. It is very likely that such a form of lignocellulosic material coated with a coupling agent will be able to have a larger surface area and more strongly interact with the elastomer matrix.

### 3.3. Contact Angle Measurement

**Figure 4:**
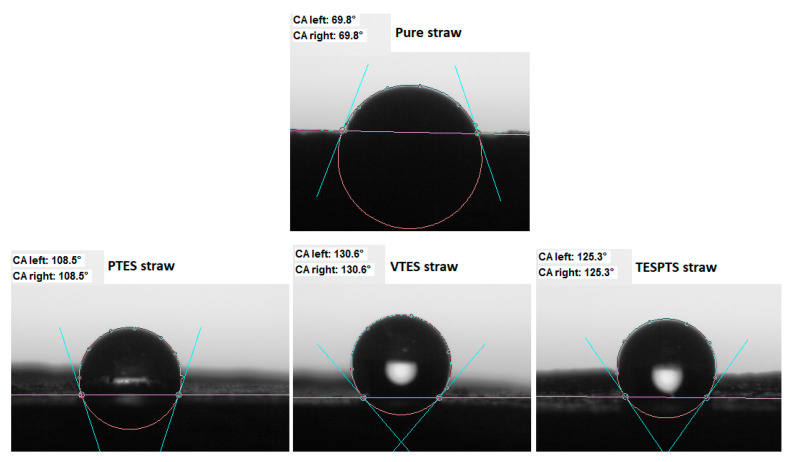
Images of drops on the surface of pure and silanized straw. CA—contact angle.

The analysis of the water wettability of the unmodified and modified straw fibers was carried out in order to evaluate the activity of the silanes used. The nature of the surface was investigated on the basis of the contact angle measurements (Figure 4).

Pure straw was characterized by the smallest contact angle, moreover, the drop was absorbed into the tested material in a very short time. The modification of the straw surface resulted in an increase in the hydrophobicity of the filler, which was reflected in the higher recorded values of the contact angle. They were PTES straw—108°, VTES straw—131°, and TESPTS straw—125°. The obtained results proved that the modifications of the materials were justified in order to use them as active fillers of non-polar natural rubber and to improve the selected functional properties of biocomposites.

### 3.4. Cure Characteristics of NR Compounds

The analysis of the data obtained on the basis of rheometric tests showed a general dependence resulting from the addition of modified natural fibers (Table 2).

In the case of all composites filled with them, the maximum torque value increased during the rheometric measurements along with the increase in the filler content, regardless of the type of modification used. Moreover, the results obtained for the modified mixtures were in each case higher compared with the reference sample and unmodified systems. Identical dependencies occurred in the case of the analysis of the minimum torque value and the increase in torque. It was undoubtedly influenced by appropriately selected modifying agents and their amounts. Most likely, as a result of the impact of silanes, PTES, VTES, and TESPTS, on straw fibers, the adhesion between the components of the mixture was improved. A consequence of the increase in filler–polymer interactions was the increase in torque, which was most probably caused by the more developed spatial structure of elastomer composites.

**Figure 5:**
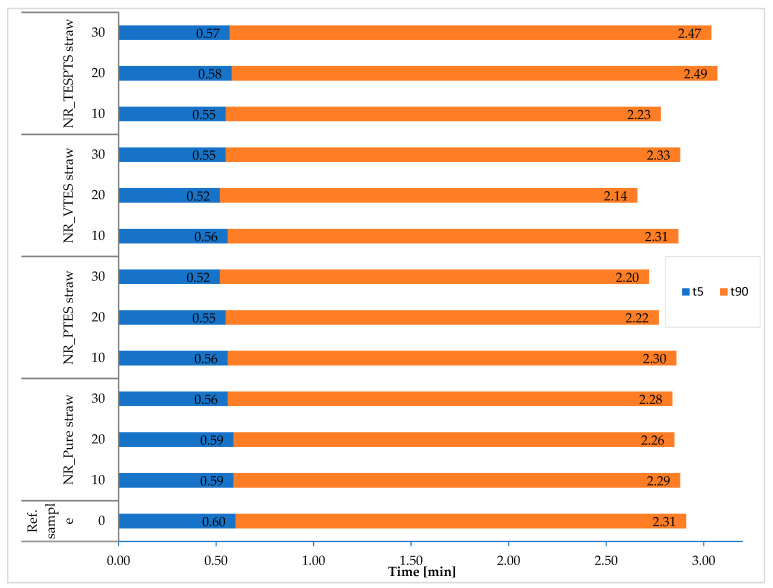
Curing characteristics of elastomer mixtures.

The conducted modifications did not have a significant impact on the vulcanization rate of elastomer blends (Figure 5).

Both the optimal vulcanization time (t_90_) and the scorch time (t_5_) for the modified composites fluctuated at similar levels to the reference sample and composites filled with pure straw. The t_90_ values ranged from 2.14 to 2.49 min and the t_5_ values ranged from 0.52 to 0.60 min. It should be emphasized, however, that the vulcanization rate of all elastomer mixtures was at a similarly low level, which is extremely advantageous from the economic point of view of processing processes.

### 3.5. Kinetics of Vulcanization of Rubber Mixtures

The effect of the straw modifications applied to the temperature and vulcanization enthalpy of natural rubber composites was investigated by means of DSC analysis (Table 3).

The process of cross-linking of natural rubber (reference sample) with a sulfur cross-linking system is an exothermic process and occurred in the temperature range of 175–188 °C, with the enthalpy of reaction equal to 7.46 J/g. The addition of straw significantly contributed to increasing the range in which the cross-linking reactions occurred. This especially affects the vulcanization start temperature, which was 152 °C for the composite filled with 10 phr of pure straw, and 149 °C for that with 30 phr. A significant effect of the pure straw application on the process enthalpy was also observed. Its value for NR_Pure straw containing 10 phr of filler increased to 12.54 J/g. The shift in the vulcanization temperature of mixtures filled with pure straw may result from the nature of the filler surface. Straw is a material that shows the alkaline character. In general, the pH level varies, depending on many factors, such as soil environment, degradation, and treatment, from about 7.5 to 9 [31,32]. As a result, it may have a positive effect on the cross-linking process. Vulcanization is more effective in an alkaline environment, thereby increasing the efficiency of the cross-linking system [33]. The silanization of the straw used as an active natural rubber additive contributed to the reduction of the process temperature by another few degrees Celsius. Perhaps, the active silane groups on the modified straw particles actively participated in the cross-linking processes, which allowed the vulcanization temperature to be exposed. The vulcanization energy effect for composites filled with silanized straw oscillated around 11–13 J/g, with the exception of a mixture containing 30 phr TESPTS straw, for which the vulcanization enthalpy was 21.07 J/g. Such a large energy effect indicated the strong activity of the cross-linking process. As a result of the modifications, there was a bound silane on the straw particles with active groups (containing additional sulfur atoms), which enhanced the cure rate by undergoing complex formation with rubber compounding ingredients, which subsequently forms crosslinks.

### 3.6. The Morphology of Straw-Filled Composites

**Figure 6:**
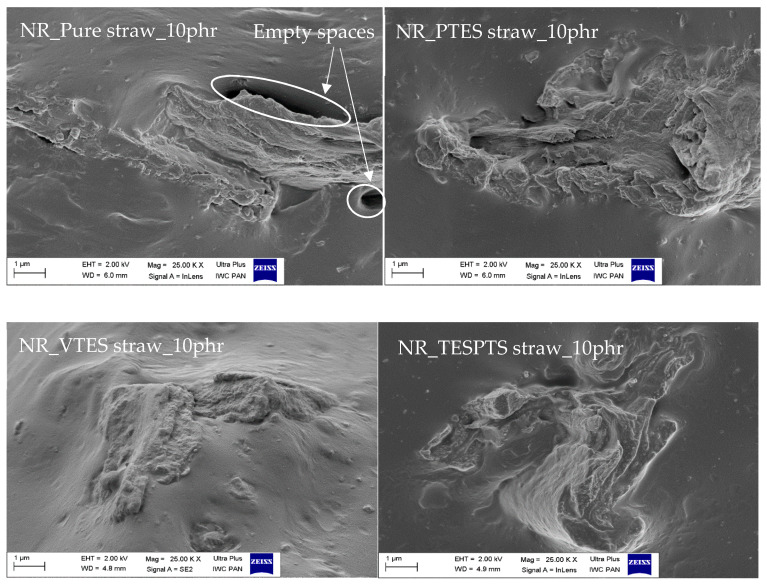
SEM images of composites with the addition of pure and modified straw (magnification × 25,000).

SEM images of the breakthroughs of the tested composites with the optimal (from the functional properties point of view) content of 10 phr are shown in Figure 6.

The SEM analysis was performed in order to determine the effect of the treatment on adhesion at the natural rubber–straw interface. The presented images clearly prove the improved compatibility of silanized straws with the elastomer matrix. On the images of the composite containing pure straw, there are visible darklings indicating the presence of voids between the rubber and the filler. As a consequence, the straw particles interact less with the polymer. In contrast, in the case of biocomposites filled with silanized fibers, better connections at the polymer–filler interface were observed. Improvement of the impacts was achieved by modification of straw, which influenced the morphology and compatibility with natural rubber. The compatibility improvement was achieved by creating a thin hydrophobic coating on the surface of the lignocellulosic material. In order to obtain the best possible adhesion, bifunctional silane particles were used, which acted as a bridge between the straw and the chain of rubber macromolecules through physical and chemical interactions between the composite components. First, by reacting the silane with hydroxyl groups present on the surface of the fibers, and then by reacting other functional groups with the polymer. As a result, an increase in intramolecular interactions in the composite is expected and, consequently, an increase in cross-linking density manifested in the reinforcement of the composite.

### 3.7. Cross-Linking Densities of Vulcanizates

**Figure 7:**
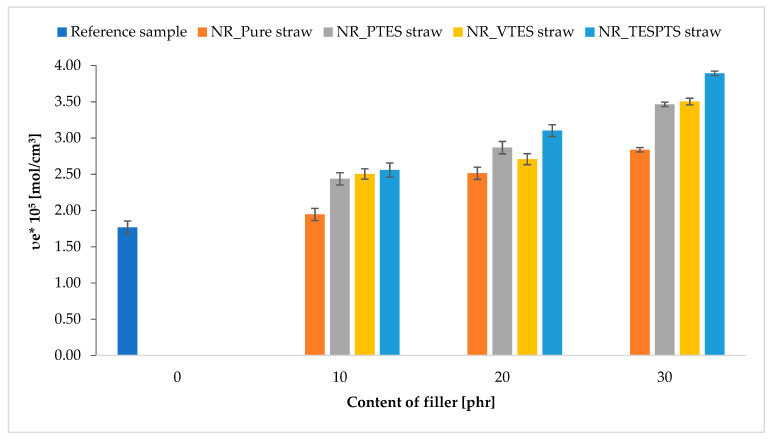
Impact of straw modifications and the fillers’ content on the cross-linking density of biocomposites.

The cross-linking density of the tested systems is presented in Figure 7.

This parameter is particularly important in the case of elastomer vulcanizates because it affects a number of functional properties of rubber products. The υ_e_ value for the tested composites was strongly dependent on the type of filler modifications and their amount.

The highest cross-linking density was characteristic for composites containing straw treated with TESPTS and slightly lower with VTES and PTES. This dependence can be explained by the different activities of the silanes used in the vulcanization process. This activity resulted from the presence of groups in the silanes that could have a different effect on the cross-linking of natural rubber. PTES does not contain groups that can actively participate in vulcanization, but nevertheless increases the hydrophobicity of the surface, and thus obtains better compatibility with natural rubber. As a result, the cross-linking density value for the NR_PTES straw composites was slightly higher than the NR_Pure straw systems and the reference sample. The vinyl double bond present in VTES is considered a vulcanization reactive site that can create additional network nodes. On the other hand, the TESPTS silane has sulfur atoms in its structure, through which cross-links, so-called sulfur bridges, between sections of the polymer chain are built. The reactive groups present on the modified straw particles could create additional connections in the spatial structure of the composite, affecting the effective number of connections formed as a result of vulcanization.

**Figure 8:**
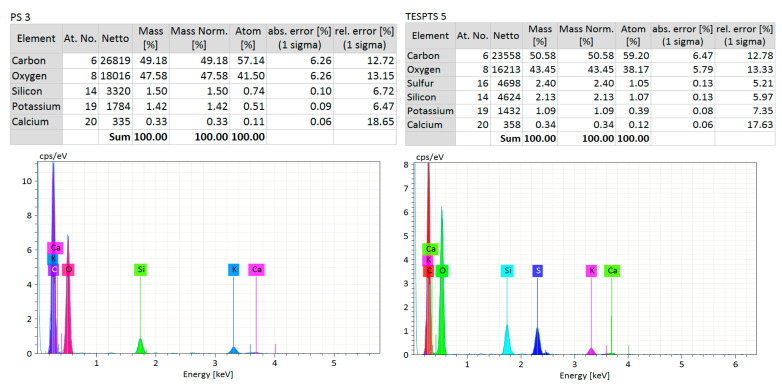
Elementary characteristic of pure straw and TESPTS straw.

The presence of additional sulfur atoms on the TESPTS modified straw particles was confirmed by the analysis of the elemental composition of the straw performed using the SEM–EDS technique (Figure 8).

The study showed that, in the TESPTS samples, apart from carbon (the quantitative value may be overestimated because of the pre-treatment with carbon before the test), oxygen, silicon, potassium, and calcium atoms also contained sulfur.

### 3.8. Mechanical Properties

**Figure 9:**
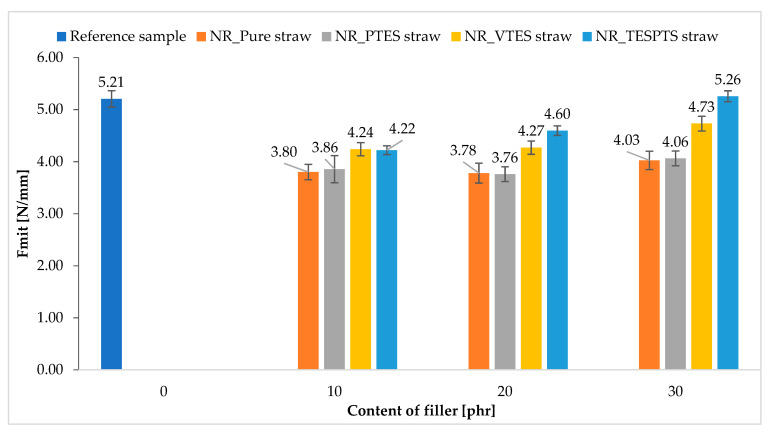
Tear resistance of natural rubber composites containing modified and unmodified straw.

The influence of the applied modifications with silanes with various functional groups can also be observed on the basis of the results of the tear strength tests of the obtained elastomer composites. The data presented in Figure 9 show that the addition of natural fillers decreased the tear resistance of natural rubber.

Nevertheless, the applied modifications with silanes significantly (even up to 30%) increased the strength of these materials. In general, the F_mit_ value increased as the filler content in the elastomer increased. The strongest influence of the modifiers was observed for the TESPTS and VTES modified vulcanizates. Improvement in the tear strength of composites may result from increased adhesion of fibers to the polymer, and thus stronger interfacial interactions influencing the increase in cross-linking density. The obtained results confirmed the previous research results and showed the highest activity of TESPTS and VTES as modifiers of straw used to reinforce natural rubber.

One of the most important functional properties of polymer composites is their mechanical strength. The results of the tensile strength tests of the obtained composites are presented in Table 4.

The addition of the natural filler to the NR resulted in a significant increase in the modules achieved at 100, 200, and 300% elongation.

These results show that straw actively strengthens the elastomer. In the case of slight deformations up to 300%, the highest modulus values were achieved for composites NR_TESPTS straw. However, for the remaining composites modified with PTES straw and VTES straw, the results of SE100, 200, and 300 are at the level similar to vulcanizates containing pure straw. The most interesting parameter in terms of mechanical properties is the tensile strength (TS). The results indicated that the straw modifications had different effects on the mechanical strength of the composites. No significant changes were observed when comparing the TS values achieved for the NR_PTES straw with the NR_Pure straw. In these composites, samples containing 20 phr of filler were characterized by higher strength. In the case of NR_VTES straw vulcanizates, TS values also oscillated at a similar level to NR_Pure straw, except for the vulcanizate filled with 20 phr straw, in which the value was higher by 2 MPa. A significant influence on the obtained tensile strength values could be observed again for vulcanizates containing straw treated with TESPTS. Once again, the positive effect of this modification on the properties of NR biocomposites was demonstrated. The obtained results are a consequence of the cross-linking growth observed for vulcanizates containing material functionalized with 3,3′-Tetrathiobis (propyl-triethoxysilane). The active influence of silane containing sulfur atoms in the vulcanization process contributed to the expansion of the composite spatial network, affecting the material strength.

Elongation at break for all vulcanizates was over 600%. Regardless of the filler used, the Eb value decreased with the increasing weight fraction of straw. The samples filled with PTES straw were characterized by the greatest elongation at the moment of breaking.

### 3.9. Thermal Stability of Composites

The thermal stability of composites containing natural fillers was assessed on the basis of the temperature, at which 5 and 50% weight loss was observed (T_5_ and T_50_). Analyzing the value of the initial decomposition temperature (T_5_), the filler addition contributed to the decrease in the thermal stability of the composites (Table 5).

The addition of a filler with low thermal stability additionally lowered the T_5_ temperature by approximately 19–38 °C compared with the reference sample. Moreover, a slight influence of a few degrees Celsius on the improvement of the thermal stability of composites containing modified straw was observed compared with NR_Pure straw vulcanizates. In the case of biocomposites containing modified straw, as a result of silanization, the adhesion between the elastomer matrix and the lignocellulosic material improved, resulting in a reduction in the volume of empty interfaces at the interface and a reduction of hydrophilic hydroxyl groups. Moreover, the silane layer formed hindered the formation of charring during thermal decomposition of straw [34]. These phenomena could have resulted in a slight improvement in the thermal stability of NR_silanized straw composites.

A thermal analysis of composites was carried out in several stages, initially in the temperature range of 25–600 °C under an inert gas atmosphere (argon). The loss of mass resulted from the pyrolysis of natural rubber, degradation of straw, and organic components included in the cross-linking unit. Consequently, with the increase in the filler content in the composite, the weight loss increased in the temperature range of 25–600 °C. Moreover, the samples containing the modified straws were characterized by a greater weight loss in the discussed temperature range, which resulted from the degradation of organosilanes used for fiber modification. Then, above 600 °C, the gas was changed to air. In this stage, the weight losses resulted from the combustion of carbon black formed in the first stage during pyrolysis. The values of weight loss ∆m (600–900 °C) were proportional to the filler content. In the case of the reference sample of natural rubber, the mineral residue after combustion (R_900_) was ash and ZnO was used as an activator. In the case of the filled composites, the residue composition was higher. This was due to the presence of ash remaining after burning the straw, which was mainly composed of silica [35].

### 3.10. Flammability of Straw

Undoubtedly, a key issue in terms of the use of lignocellulosic raw materials as bio-fillers for polymers is the radical limitation of their flammability. The literature review shows that the flammability of natural fibers largely depends on their chemical composition. Even a small amount of lignin (10%) increases the resistance of natural fibers to fire. The research carried out by Kozłowski and colleagues clearly shows that natural fibers rich in lignin (flax, hemp) are characterized by a lower rate of heat released than fibers with a high cellulose content. This shows the ability of lignin to catalyze carbonization reactions, which positively affect the formation of an insulating carbon layer, increasing the fire resistance of polymer bio-composites containing them [19,20].

The increase in the carbonization reaction efficiency during the decomposition of natural fibers can be obtained by subjecting them to chemical modification. In the subject literature, it is possible to find a few articles on fire retarding natural fibers. For example, Kondola and Horrocks modified cellulose (viscose) as well as cellulose fabric using both melamine and phosphor flame retardants. On the basis of the conducted research, they found that a significant amount of carbon generated above 500 °C improves the thermal properties of the modified fibers, as evidenced by both the increase in their activation energy and the reduction of thermal conductivity [21]. Flambard et al. investigated flame-retardant flax and wool, intended for the production of fabrics used in public transport, mixing them with PPTA (poly (p-phenylenediamine terephthalamide). The modified fibers were characterized not only by high resistance to fire, but also resistance to the action of UV light, satisfactory mechanical parameters, biodegradability, and easy processing [22]. Suardana et al. modified coir and jute fibers using diammonium phosphate (DAP) and then incorporated them into a thermoplastic polymer matrix. On the basis of the tests performed, they found that the improvement of the bio-composites’ resistance to fire was directly proportional to the amount of DAP fibers used for modification, which resulted directly from the amount of carbonaceous carbon produced above 500 °C [23]. There is no information in the literature on the subject of chemical modification of lignocellulosic fibers with silicon compounds.

The analysis of the flammability of the lignocellulosic filler showed that its chemical modification with the use of silane compounds led to a slight reduction of its flammability, expressed by the following parameters: maximum heat release rate (HRR), total heat released (THR), and heat capacity (HRC) (Table 6).

The greatest reduction of the HRR parameter was recorded for 3,3′-tetrathiobis (propyl-triethoxysilane) (TESPTS). The wheat straw modified with the use of TESPTS was also characterized by the lowest rate of thermal decomposition (DTG_TESPTS curve) (Figure 2). It should be clearly emphasized that the slight reduction in flammability of natural fibers modified with silicon compounds was a consequence of an increase in their thermal stability expressed by the parameter of a 5% loss in sample mass (VTES straw and TESPTS straw) (Figure 2). The applied modification had a marginal effect on the intensification of carbonization processes, parameter R_600_ (Figure 2).

### 3.11. Flammability of Selected NR Composites

The introduction of unmodified wheat straw into the natural rubber matrix significantly reduced its flammability. Only 10 parts wt. the filler used (pure straw) significantly reduced the value of the HRR, HRRmax, THR, and EHC parameters. It should also be noted that the mass loss rate (MLR), HRR_max_/tHRR_max_ (FIGRA), and maximum average heat release rate (MARHE) parameters, directly indicating the intensity of the combustion process, and thus the fire development, were also significantly reduced (Table 7).

**Figure 10:**
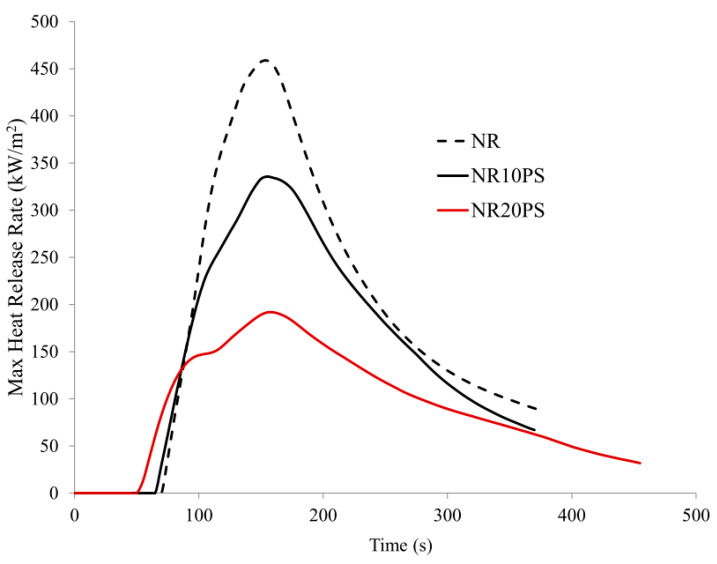
HRR_max_ curves of NR rubber composites containing pure straw filler.

**Figure 11:**
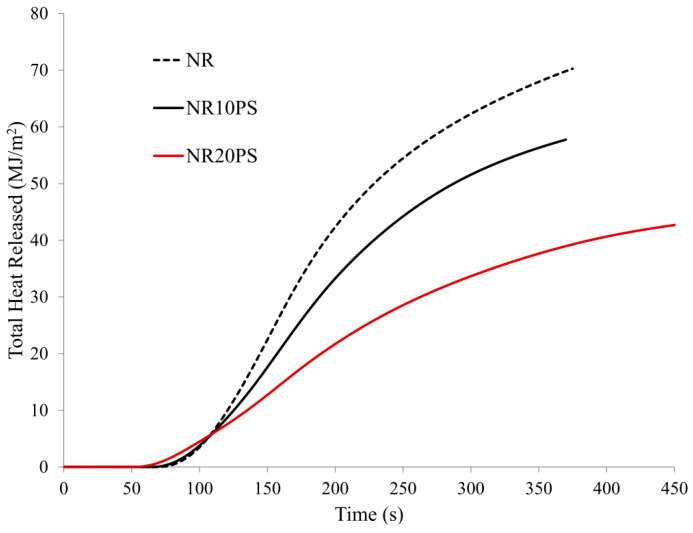
Total heat release (THR) curves of NR rubber composites containing pure straw filler.

Increasing the content of unmodified natural filler (pure straw) to 20 parts. wt in a natural rubber matrix (sample NR20PS) resulted in a reduction of the HRR_max_ parameter by as much as 58% concerning the cross-linked, unfilled NR rubber (NR sample) (Table 7, Figure 10 and Figure 11).

The THR (39%), EHC (43.7%), FIGRA (59.6%), and MARHE (47.6%) parameters were also significantly reduced.

The reduction in the flammability of composites containing an unmodified lignocellulosic filler, that is, carbon filler, was directly related to the combustion process. A feedback mechanism takes place during the combustion process of composites containing the unmodified natural filler. The lignin-rich cellulose undergoes a stepwise, rapid thermal decomposition during the combustion of the sample. Each stage of the filler decomposition (the so-called microblash, which is visible during the flammability test) is accompanied by the absorption of large amounts of oxygen, an increase in the optical density of smoke and carbon, and consequently the extinction of the flame. The presented flammability reduction mechanism is confirmed by the parameters of AMLR, FIGRA, and MARHE.

**Figure 12:**
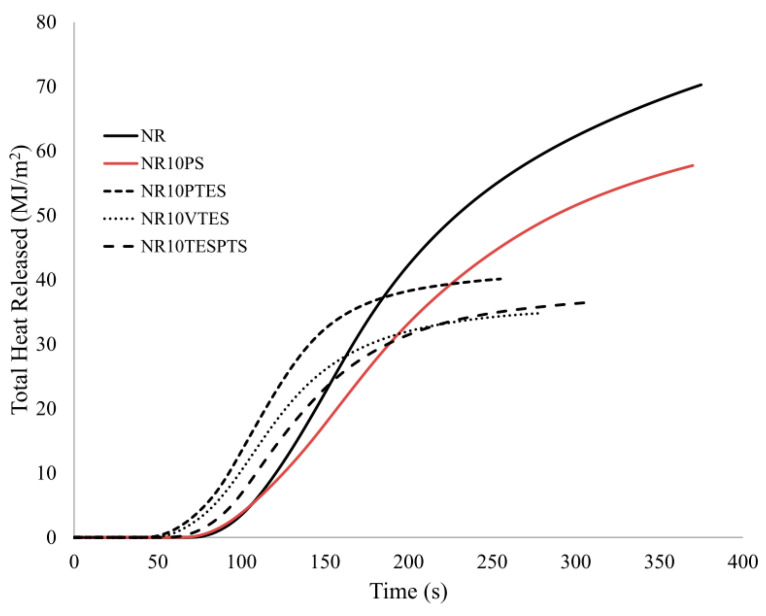
THR curves of NR rubber composites containing unmodified and modified natural filler.

The modification of wheat straw with silicon compounds had an ambiguous effect on the reduction of the flammability of the natural rubber composites containing them. On the one hand, the use of a modified filler eliminated the feedback mechanism, while on the other hand, it should be noted that the modification of pure straw with silane compounds significantly reduced the value of the THR or EHC parameter (Table 7, Figure 12).

In the case of the TESPTS silane modification, a reduction in the value of the HRR parameter and HRR_max_ was also found (sample NR10PS vs. NR10TESPTS). It should be noted that the reduction of the fire hazard parameters of the samples containing the modified filler as compared with the samples containing the unmodified filler resulted mainly from the better dispersion of silanized cellulose in the NR rubber matrix.

## 4. Conclusions

Modifications of lignocellulosic fibers in the form of ground cereal straw with propyltriethoxysilane, vinyltriethoxysilane, and 3,3′-tetrathiobis (propyl-triethoxysilane) significantly influenced both the characteristics of the fillers and the properties of the obtained biocomposites.

In the case of modified lignocellulosic materials, the following was noted:

- Improvement of the thermal stability of natural additives, in particular for VTES and TESPTS straw.
- Increase in the hydrophobicity of the fillers’ surface (almost twofold increase in the value of the contact angle in comparison with the unmodified sample). The change like the surface contributed to a better compatibility of the filler with the elastomer matrix.
- Change of the surface morphology of natural fibers. After the modification, the straw structure was less smooth and more dispersed. The fibers were characterized by heterogeneity in terms of size and shape.

The general conclusions about natural rubber composites filled with modified cereal straw were as follows:

- The vulcanizates containing silanized natural fibers showed an increase in torque gain recorded during rheometric tests. The highest value of ΔM was characteristic for composites containing straw treated with VTES and TESPTS, which resulted in increased cross-linking density. This phenomenon was influenced by the modification of the fibers with silanes, which had active groups in their structure that could participate in the vulcanization process. Both the additional sulfur atoms found in the TESPTS structure and the double bonds derived from VTES present on the surface of the modified straw played an important role in the interaction at the polymer–filler interface and contributed to the development of the spatial network of composites. The activity of these modifications in the vulcanization process was confirmed by DSC analysis, which showed that the vulcanization start temperature of the mixtures filled with silanized straws was lower and the thermal effect was higher compared with the reference systems.
- As a consequence of the increase in cross-linking density and the improvement of the compatibility of the modified fibers with natural rubber (analysis of SEM photos), the vulcanizates were also characterized by better mechanical properties. Evidence of these changes was the increased tensile strength compared with the system containing the unmodified filler and the reference test.
- As a result of the improvement of the adhesion between the components of biocomposites (natural rubber–modified straw), the reduction of void volumes at the interface, and the reduction of hydroxyl groups, a slight improvement in the thermal stability of composites containing silanization straw was also observed with the NR_Pure straw systems.
- The modification of cereal straw with silanes had a different effect on the flammability of biocomposites. The use of a modified filler eliminates the feedback mechanism, while reducing the values of THR and EHC parameters. Treatment with 3,3′-tetrathiobis (propyl-triethoxysilane) also lowered the HRR and HRR_max_ parameters compared with the composite filled with pure straw.

## Figures and Tables

**Table 1:** Silane structures.

Silane	Silane Structure
**n-Propyltriethoxysilane**	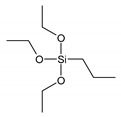
**Vinyltriethoxysilane**	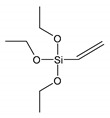
**3,3′-Tetrathiobis(propyl-triethoxysilane)**	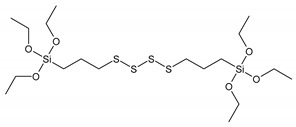

**Table 2:** Rheometric properties of natural rubber composites filed with: -pure straw (NR_Pure straw); -straw modified with propyltriethoxysilane (NR_PTES straw); -straw modified with vinyltriethoxysilane (NR_VTES straw); -straw modified with 3,3′-Tetrathiobis(propyl-triethoxysilane) (NR_TESPTS straw).

Reference Sample	Content of Filler [phr]	ML [dNm]	MH [dNm]	∆M [dNm]
	0	0.60	5.44	4.84
NR_Pure straw	10	0.65	6.10	5.45
	20	0.78	7.16	6.38
	30	0.85	7.59	6.74
NR_PTES straw	10	0.75	6.35	5.60
	20	0.85	7.32	6.47
	30	0.95	8.62	7.67
NR_VTES straw	10	0.82	6.51	5.69
	20	0.95	8.42	7.47
	30	1.02	9.40	8.38
NR_TESPTS straw	10	0.70	6.45	5.75
	20	0.80	7.52	6.72
	30	1.00	8.73	7.73

ML—minimum torque; MH—maximum torque, ΔM—torque gain during cross-linking.

**Table 3:** Vulcanization kinetics parameters of rubber mixtures obtained on the basis of differential scanning calorimetry (DSC) analysis.

Sample	Content of Filler [phr]	T_onset_ [°C]	T_endset_ [°C]	Q_p_ [J/g]
Ref. Sample	0	176	188	7.46
NR_Pure straw	10	152	189	12.54
NR_PTES straw		145	214	12.28
NR_VTES straw		137	219	13.26
NR_TESPTS straw		145	218	12.72
NR_Pure straw	30	149	212	12.49
NR_PTES straw		149	211	11.93
NR_VTES straw		143	217	11.66
NR_TESPTS straw		147	223	21.07

T_onset_—initial temperature of the cross-linking process; T_endset_—end temperature of the cross-linking process; Q_p_—thermal effect of the cross-linking process.

**Table 4:** Mechanical properties of NR biocomposites.

Sample	Content of Filler [phr]	SE_100_ [MPa]	SE_200_ [MPa]	SE_300_ [MPa]	TS [MPa]	Eb [%]
Reference sample	0	0.75	1.12	1.53	12.8	680
NR_Pure straw	10	1.44	2.01	2.54	15.3	696
	20	1.50	2.28	3.08	15.9	651
	30	2.00	2.96	3.14	13.1	622
NR_PTES straw	10	1.01	1.62	2.17	14.9	727
	20	1.37	1.75	2.81	15.2	688
	30	1.73	2.58	3.32	13.5	660
NR_VTES straw	10	1.03	1.63	2.19	14.2	668
	20	1.08	1.75	2.38	17.9	656
	30	1.76	2.56	3.26	13.6	649
NR_TESPTS straw	10	1.05	1.80	2.61	17.0	699
	20	1.35	2.39	3.41	16.7	673
	30	1.91	3.22	4.31	14.3	612

SE_100, 200, 300_—modules achieved at 100, 200, and 300% elongation; TS—tensile strength; Eb—elongation at break.

**Table 5:** Influence of straw modifications on the thermal stability of biocomposites.

Sample	Content of Filler [phr]	T_5_ [°C]	T_50_ [°C]	∆m (25–600 °C) [%]	∆m (600–900 °C) [%]	R_900_ [%]
Ref. sample	0	319	389	6.2	2.3	3.9
NR_Pure straw	10	290	384	8.9	3.9	5.0
	20	285	383	8.8	3.6	5.1
	30	281	381	9.0	3.6	5.4
NR_PTES straw	10	299	385	7.2	2.4	4.8
	20	289	384	8.1	3.1	5.0
	30	283	384	10.6	4.8	5.8
NR_VTES straw	10	299	387	7.3	2.5	4.7
	20	300	385	7.4	2.6	4.9
	30	285	382	8.7	3.7	5.0
NR_TESPTS straw	10	295	383	7.2	2.6	4.6
	20	285	380	8.0	3.1	4.9
	30	285	383	9.1	3.9	5.2

**Table 6:** Flammability results of unmodified and chemically modified cereal straw.

Sample	HRR [W/g]	THRR [°C]	THR [kJ/g]	HRC [J/gK]
Pure straw	185	338	17.4	195
PTES straw	167	335	10.9	164
VTES straw	164	341	10.9	161
TESPT straw	149	336	11.8	146

HRR—maximum heat release rate; THRR—temperature of the maximum heat release rate; THR—total heat released; HRC—heat capacity.

**Table 7:** Flammability results of composites containing unmodified and modified natural filler.

Combustibility Parameters	Vulcanized Composites Description					
	NR	NR_10PS	NR_20PS	NR_10PTES	NR_10VTES	NR_10TESPTS
t_i_ (s)	60	54	38	64	44	51
t_f-o_ (s)	287	296	337	249	187	248
HRR (kW/m^2^)	264.3	209.6	122.4	214.5	220.1	178.4
HRR_max_ (kW/m^2^)	458.5	335.6	191.6	448.3	367.3	371.03
tHRR_max_ (s)	155	155	160	140	110	110
THR (MJ/m^2^)	60.2	51.0	36.5	39.5	31.4	34.6
EHC (MJ/kg)	33.4	26.4	18.8	20.7	16.23	17.72
EHC_max_ (MJ/kg)	56.9	76.5	77.5	66.7	63.09	77.05
MLR (g/s)	0.07	0.07	0.05	0.09	0.12	0.08
MLR_max_ (g/s)	0.265	0.249	0.257	0.241	0.247	0.269
AMLR (g/m^2^s)	18.21	14.81	14.28	19.12	19.93	18.48
FIGRA (kW/m^2^s)	2.95	2.16	1.19	3.20	3.33	3.37
MARHE (kW/m^2^)	218.3	176.8	114.2	215.8	173.9	161.3
Burning droplets	no	yes	yes	no	no	no

t_i_—time to ignition; t_f-o_—time to flameout; HRR—heat release rate; HRR_max_—maximum heat release rate; tHRR_max_—time to maximum heat release rate; THR—total heat release; EHC—effective heat of combustion; EHC_max_—maximum effective heat of combustion; MLR—mass loss rate; MLR_max_—maximum mass loss rate; AMLR—average mass loss rate; FIGRA—HRR_max_/tHRR_max_; MARHE—maximum average heat release rate.

## References

[1] Arrakhiz F.Z., El Achaby M., Malha M., Bensalah M.O., Fassi-Fehri O., Bouhfid R., Benmoussa K., Qaiss A. (2013). Mechanical and thermal properties of natural fibers reinforced polymer composites: Doum/low density polyethylene. Mater. Des..

[2] Di Bella G., Fiore V., Galtieri G., Borsellino C., Valenza A. (2014). Effects of natural fibres reinforcement in lime plasters (kenaf and sisal vs. Polypropylene). Constr. Build. Mater..

[3] Ku H., Wang H., Pattarachaiyakoop N., Trada M. (2011). A review on the tensile properties of natural fiber reinforced polymer composites. Compos. Part B Eng..

[4] Verma D., Senal I. (2019). Natural fiber-reinforced polymer composites. Biomass Biopolym. Mater. Bioenergy.

[5] Faruk O., Bledzki A.K., Fink H.-P., Sain M. (2012). Biocomposites reinforced with natural fibers: 2000–2010. Prog. Polym. Sci..

[6] Mohammed L., Ansari M.N.M., Pua G., Jawaid M., Islam M.S. (2015). A Review on Natural Fiber Reinforced Polymer Composite and Its Applications. Int. J. Polym. Sci..

[7] Nair A.B., Joseph R. (2014). Eco-friendly bio-composites using natural rubber (NR) matrices and natural fiber reinforcements. Chemistry, Manufacture and Applications of Natural Rubber.

[8] Moran-Mirabal J.M. (2013). The study of cell wall structure and cellulose–cellulase interactions through fluorescence microscopy. Cellulose.

[9] Saleem Khan T., Mubeen U. (2012). Wheat Straw: A Pragmatic Overview. Curr. Res. J. Biol. Sci..

[10] Rahman K. (2017). Evaluating the Potential of Rice Straw as a Co-digestion Feedstock for Biogas Production in Bangladesh. J. Adv. Catal. Sci. Technol..

[11] Panthapulakkal S., Zereshkian A., Sain M. (2006). Preparation and characterization of wheat straw fibers for reinforcing application in injection molded thermoplastic composites. Bioresour. Technol..

[12] Ansarifar A., Wang L., Ellis R.J., Kirtley S.P., Riyazuddin N. (2007). Enhancing the mechanical properties of styrene–butadiene rubber by optimizing the chemical bonding between silanized silica nanofiller and the rubber. J. Appl. Polym. Sci..

[13] Szadkowski B., Marzec A., Rybiński P. (2020). Silane Treatment as an Effective Way of Improving the Reinforcing Activity of Carbon Nanofibers in Nitrile Rubber Composites. Materials.

[14] Roy K., Debnath S.C., Tzounis L., Pongwisuthiruchte A., Potiyaraj P. (2020). Effect of Various Surface Treatments on the Performance of Jute Fibers Filled Natural Rubber (NR) Composites. Polymers.

[15] Srisuwan L., Jarukumjorn K., Suppakarn N. (2018). Effect of Silane Treatment Methods on Physical Properties of Rice Husk Flour/Natural Rubber Composites. Adv. Mater. Sci. Eng..

[16] Ismail H., Othman N., Komethi M. (2012). Curing characteristics and mechanical properties of rattan-powder-filled natural rubber composites as a function of filler loading and silane coupling agent. J. Appl. Polym. Sci..

[17] Cabrera Álvarez E.N., Ramos-deValle L.F., Sánchez-Valdes S., Ramírez-Vargas E., Espinoza-Martinez A.B., Rodriguez-Fernandez O.S., Beltran-Ramırez F.I., Méndez Nonell J., Sanchez-Cuevas J.L. (2017). Study of the Effect of a Polymeric Compatibilizing Agent on the Flame Retardancy and Tensile Properties of High Density Polyethylene/Magnesium Hydroxide Compositions. Macromol. Symp..

[18] Rybiński P., Janowska G. (2013). Flammability and other properties of elastomeric materials and nanomaterials. Part I. Nanocomposites of elastomers with montmorillonite or halloysite. Polimery.

[19] Kozłowski R., Władyka-Przybylak M. (2008). Flammability and fire resistance of composites reinforced by natural fibers. Polym. Adv. Technol..

[20] Kozlowski R., Przybylak M.W. (2001). Natural polymers, wood and lignocellulosic materials. Fire Retardant Materials.

[21] Kandola B.K., Horrocks A.R. (2000). Complex char formation in flame-retarded fibre-intumescent combinations? IV. Mass loss and thermal barrier properties. Fire Mater..

[22] Flambard X., Bourbigot S., Kozlowski R., Muzyczek M., Mieleniak B., Ferreira M., Vermeulen B., Poutch F. (2005). Progress in safety, flame retardant textiles and flexible fire barriers for seats in transportation. Polym. Degrad. Stab..

[23] Suardana N.P.G., Ku M.S., Lim J.K. (2011). Effects of diammonium phosphate on the flammability and mechanical properties of bio-composites. Mater. Des..

[24] Masłowski M., Miedzianowska J., Strzelec K. (2017). Natural rubber biocomposites containing corn, barley and wheat straw. Polym. Test..

[25] Masłowski M., Miedzianowska J., Strąkowska A., Strzelec K., Szynkowska M.I. (2018). The use of rye, oat and triticale straw as fillers of natural rubber composites. Polym. Bull..

[26] Masłowski M., Miedzianowska J., Strzelec K. (2019). Silanized cereal straw as a novel, functional filler of natural rubber biocomposites. Cellulose.

[27] Flory P.J., Rehner J. (1943). Statistical Mechanics of Cross-Linked Polymer Networks I. Rubberlike Elasticity. J. Chem. Phys..

[28] Halvorson R.H., Erickson R.L., Davidson C.L. (2003). The effect of filler and silane content on conversion of resin-based composite. Dent. Mater..

[29] Bilba K., Arsene M.-A. (2008). Silane treatment of bagasse fiber for reinforcement of cementitious composites. Compos. Part A Appl. Sci. Manuf..

[30] Gañan P., Garbizu S., Llano-Ponte R., Mondragon I. (2005). Surface modification of sisal fibers: Effects on the mechanical and thermal properties of their epoxy composites. Polym. Compos..

[31] Ward W.R., Hughes J.W., Faull W.B., Cripps P.J., Sutherland J.P., Sutherst J.E. (2002). Observational study of temperature, moisture, pH and bacteria in straw bedding, and faecal consistency, cleanliness and mastitis in cows in four dairy herds. Vet. Rec..

[32] Halvarsson S., Edlund H., Norgren M. (2010). Wheat straw as raw material for manufacture of medium density fiberboard (MDF). BioResources.

[33] Maciejewska M., Sowińska A. (2019). Thermal characterization of the effect of fillers and ionic liquids on the vulcanization and properties of acrylonitrile–butadiene elastomer. J. Therm. Anal. Calorim..

[34] Wladyka-Przybylak M., Wesolek D., Rojewski S., Gasiorowski R., Gieparda W., Bujnowicz K., Maciejewski H., Wojcik R., Nowicki M. (2015). Synergistic Effect of Modified Natural Fibres with Halogen-Free Fire Retardants in Reducing Flammability of Composites. J. Biobased Mater. Bioenergy.

[35] Dodson J.R., Hunt A.J., Budarin V.L., Matharu A.S., Clark J.H. (2011). The chemical value of wheat straw combustion residues. RSC Adv..

